# Unveiling macrophage diversity in myocardial ischemia-reperfusion injury: identification of a distinct lipid-associated macrophage subset

**DOI:** 10.3389/fimmu.2024.1335333

**Published:** 2024-02-21

**Authors:** Ying Jiang, Wenpeng Yu, Tie Hu, Hanzhi Peng, Fajia Hu, Yong Yuan, Xufeng Liu, Songqing Lai, Jianliang Zhou, Xiao Dong

**Affiliations:** ^1^ Department of Cardiovascular Surgery, The Second Affiliated Hospital, Jiangxi Medical College, Nanchang University, Nanchang, China; ^2^ Department of Cardiovascular Surgery, The First Affiliated Hospital, Jiangxi Medical College, Nanchang University, Nanchang, China; ^3^ Department of Haematology, Ganzhou People’s Hospital, Ganzhou, China; ^4^ Department of Cardiovascular Surgery, Zhongnan Hospital of Wuhan University, Wuhan, China

**Keywords:** myocardial ischemia-reperfusion injury, single-cell sequencing, bioinformatics, macrophage heterogeneity, lipid-associated macrophage

## Abstract

**Background and objective:**

Macrophages play a crucial and dichotomous role cardiac repair following myocardial ischemia-reperfusion, as they can both facilitate tissue healing and contribute to injury. This duality is intricately linked to environmental factors, and the identification of macrophage subtypes within the context of myocardial ischemia-reperfusion injury (MIRI) may offer insights for the development of more precise intervention strategies.

**Methods:**

Specific marker genes were used to identify macrophage subtypes in GSE227088 (mouse single-cell RNA sequencing dataset). Genome Set Enrichment Analysis (GSEA) was further employed to validate the identified LAM subtypes. Trajectory analysis and single-cell regulatory network inference were executed using the R packages Monocle2 and SCENIC, respectively. The conservation of LAM was verified using human ischemic cardiomyopathy heart failure samples from the GSE145154 (human single-cell RNA sequencing dataset). Fluorescent homologous double-labeling experiments were performed to determine the spatial localization of LAM-tagged gene expression in the MIRI mouse model.

**Results:**

In this study, single-cell RNA sequencing (scRNA-seq) was employed to investigate the cellular landscape in ischemia-reperfusion injury (IRI). Macrophage subtypes, including a novel Lipid-Associated Macrophage (LAM) subtype characterized by high expression of Spp1, Trem2, and other genes, were identified. Enrichment and Progeny pathway analyses highlighted the distinctive functional role of the SPP1+ LAM subtype, particularly in lipid metabolism and the regulation of the MAPK pathway. Pseudotime analysis revealed the dynamic differentiation of macrophage subtypes during IRI, with the activation of pro-inflammatory pathways in specific clusters. Transcription factor analysis using SCENIC identified key regulators associated with macrophage differentiation. Furthermore, validation in human samples confirmed the presence of SPP1+ LAM. Co-staining experiments provided definitive evidence of LAM marker expression in the infarct zone. These findings shed light on the role of LAM in IRI and its potential as a therapeutic target.

**Conclusion:**

In conclusion, the study identifies SPP1+ LAM macrophages in ischemia-reperfusion injury and highlights their potential in cardiac remodeling.

## 1 Introduction

Myocardial infarction (MI) is a leading cause of morbidity and mortality related to cardiovascular diseases (1). The mainstay of treatment involves the appropriate and timely restoration of blood flow through antithrombolytic drugs or mechanical interventions (2). However, despite successful blood flow restoration, abrupt recanalization can further exacerbate myocardial damage, resulting in cardiomyocyte death, a condition referred to as myocardial ischemia-reperfusion injury (IRI) (3). This phenomenon involves complex mechanisms, in which macrophages play a crucial role in regulating cardiac inflammation, with a dual function of contributing to tissue repair and potentially accelerating myocardial damage (4, 5).

In both humans and mice, cardiac macrophages can primarily be distinguished based on CCR2 (C-C chemokine receptor type 2) expression: resident CCR2- and recruited CCR2+ macrophages originate from subpopulations of embryonic and hematopoietic origin, respectively (6, 7). CCR2- and CCR2+ macrophages serve distinct functions in the heart. CCR2- macrophages are involved in various forms of tissue remodeling, including coronary artery development, vasodilatation, and cardiac tissue repair (8). On the other hand, CCR2+ macrophages become activated and promote inflammation during myocardial injury. A study conducted by Geetika et al. (9) demonstrated that CCR2+ macrophages within the tissue become activated following cardiomyocyte death. They may play a role in the initial recruitment of monocytes to the injured heart through the production of chemokines and may influence their differentiation into a population of inflammatory monocyte-derived macrophages. Furthermore, these derived monocyte populations may undergo further differentiation into various subtypes to fulfill their distinct biological functions.

Recent advancements in macrophage biology have focused attention on a new subtype, termed lipid-associated macrophages (LAM) (10), which has sparked considerable interest. Notably, in atherosclerosis, they are identified as TREM2-high foamy macrophages (11). In the context of colorectal cancer (CRC), they potentially correspond to SPP1+ tumor-associated macrophages (12). Furthermore, in triple-negative breast cancer (TNBC), LAMs are classified as a STAB1+TREM2+ high lipid-associated macrophage subgroup (13). Although a consensus has not yet been reached, an increasing body of research supports the hypothesis that LAM primarily have a protective function (10, 14–16). Characterized by high metabolic activity, they play a crucial role in anti-inflammatory responses and tissue remodeling (14, 17). This emerging understanding underscores the multifaceted and significant role of LAM in pathology, surpassing traditional concepts of macrophage functionality.

In this study, we utilized single-cell RNA sequencing (scRNA-seq) data to investigate the diversity of murine cardiac macrophages in the context of ischemia-reperfusion injury (IRI). We identified a minimum of five distinct macrophage subpopulations, and notably, our study revealed the presence of SPP1+ lipid-associated macrophages (LAM) within myocardial ischemia-reperfusion injury. These LAM populations exhibited striking similarities to foamy macrophages observed in models of atherosclerosis and neurodegenerative diseases (18, 19). These findings collectively underscore the diversity of macrophage populations within the context of MIRI and provide characterization of a previously unreported subpopulation of lipid-associated macrophages.

## 2 Materials and methods

### 2.1 Collection of online datasets

From the GEO (Gene Expression Omnibus) database (20), we conducted searches utilizing the keywords “myocardial ischemia-reperfusion injury” to retrieve relevant transcriptomic datasets associated with MIRI. The selected datasets are as follows:

GSE227088 (21) (single-cell transcriptome sequencing): Comprises 9 samples, with 6 samples from both the Sham group and the MIRI group chosen for inclusion in this research.

GSE145154 (22) (single-cell transcriptome sequencing): A dataset consisting of 6 heart failure samples resulting from ischemic cardiomyopathy (ICM) and 2 control samples was selected for validation of human specimens.

For detailed information on these datasets, please refer to **Table 1**.

**Table 1:** Basic details about the GEO dataset used in this research.

GSE series	Organism	tissue	Sample size		Platform
			Sham	IRI	
GSE227088	Mus musculus	heart	3	3	GPL24247
			Control	ICM	
GSE145154	Homo sapiens	heart	2	6	GPL20795

GEO, Gene expression omnibus; IRI, Ischemia-reperfusion injury; ICM, Ischemic cardiomyopathy.

### 2.2 Mouse myocardial ischaemia-reperfusion injury model

C57/BL6 mice (male, 8-10 weeks old) were purchased from Nanchang University Laboratory Animal Center. All animal experiments were approved and conducted in strict accordance with the guidelines of the Institutional Animal Care and Use Committee of Nanchang University. Mice were randomized into two groups (n = 10): 1) sham-operated group (control group), and 2) ischemia-reperfusion (model group). Myocardial ischemia-reperfusion injury was modeled by ligating the left anterior descending branch (LAD) of the coronary artery and performing recanalization. Briefly, mice were anesthetized with 4% isoflurane, tracheally intubated and connected to a small animal ventilator having 1.5% isoflurane. An electrocardiogram was used to monitor the mice in real time during the experiment to ensure that the experimental model was competent. A left intercostal thoracotomy was performed on mice to induce a fourth intercostal IRI and expose the heart. Ligation was performed by binding the LAD and PE-10 sterile tubes together with 10-0 nylon sutures, which could block coronary blood flow and avoid irreversible damage to blood vessels and myocardium. The tubes were removed after 30 minutes of ischemia. Observation (anterior ventricular wall becoming pale) and ECG monitoring (ST-T segment elevation) were used to determine the success of coronary occlusion, and reversal of these effects was a sign of successful reperfusion. sham group underwent the same surgical procedure without LAD ligation and recanalization. Penicillin sodium (80,000 U per mouse) was injected intraperitoneally postoperatively to prevent postoperative infection. The reperfusion procedure lasted 24 hours. Both groups were sampled 24 hours after reperfusion (21).

### 2.3 Conventional analysis of single-cell data

The mouse single-cell sequencing dataset, GSE227088, includes three sham groups and three IRI groups. Data analysis was conducted using the R package Seurat (23, 24) v4.3.0, which involved pre-data quality control criteria (nCount_RNA<6000 and nFeature_RNA<4000). The data was then normalized using the NormalizeData function. Subsequently, the FindVariableFeatures function was employed to identify the top 2000 genes with the highest variance, and data scaling was performed using the ScaleData function. Dimensionality reduction was carried out through principal component analysis (PCA) using the first 30 principal components. Batch effect correction was performed using Harmony (25) v0.1.1. For data visualization, t-distributed stochastic neighbor embedding (t-SNE) plots were used to represent the first 20 principal components. Nearest-neighbor searches and clustering were conducted using the FindNeighbors and FindClusters functions, applying a resolution setting of 0.8 to achieve an optimal balance of cluster granularity. Cell cluster annotation was performed using the computational tool SingleR (26) v2.2.0, which employs a comparison between MouseRNAseqData, an annotated reference dataset, and selected marker genes to effectively distinguish cellular phenotypes (26). Manual annotation was performed based on the biological background provided by Yamada (27) and others, relying on the available biological context, as well as markers identified in cellmarker 2.0 (28). Cell counts were computed for each cell type within their respective categories. Subsequently, percentage columns were generated to calculate the proportion of cell types within each group. A subset of data for the relevant cell type was extracted from the raw data using the subset function, and differentially expressed genes were identified using the FindAllMarkers function for the current subset of data. Differentially expressed genes were determined using the Wilcoxon test with a log-fold change threshold set to 0.5, based on different cell clusters.

### 2.4 Macrophage subtype identification

Annotated macrophages were selected from the overall cell population, and new Seurat objects were created using these selected cells. Highly variable genes (HVGs) were then determined, and the expression matrix was subsequently scaled and normalized. Subsequently, shared nearest neighbor (SNN) plots were generated based on the first 5 principal components. The resolution parameter was fine-tuned to achieve a macrophage cluster number ranging from 5 to 10. Resident and recruited macrophage populations were identified based on established marker gene expression patterns, guided by available biological background. Distinctions were made between CCR2+ recruited macrophages and CCR2- resident macrophages. Classification of macrophage clusters encompassed resident macrophages, MoMF (monocyte-derived macrophage), and various differentiated subtypes. Possible macrophage types were characterized according to specific markers, as indicated in (29). For instance, resident macrophages were distinguished by the expression of Lyve1, Cd163, and Ccl24, whereas MoMF were defined by the presence of Ccr2 and the expression of antigen-processing/presenting genes (MHC-II genes: H2-Aa, H2-Ab1, H2-Eb1, H2-DMa, H2-DMb1, and Cd74). To visualize macrophage subtypes, as well as their distribution and expression within groups, several visualization R packages were employed. These include scRNAtoolVis v0.0.4, Nebulosa (30) v1.10.0, and the plot1cell.

### 2.5 Enrichment and pathway analysis

We utilized the FindAllMarkers function from the R package Seurat to identify marker genes for each cell cluster based on statistical tests and screening criteria. Gene annotation and pathway enrichment resources were accessed through org.Mm.eg.db and clusterProfiler (31), both R packages. Enrichment analyses were conducted using the enrichCluster function, focusing on clusters of macrophage subtypes. These analyses involved different types of terms from the Gene Ontology (GO) (32), specifically biological process (BP), cellular component (CC), molecular function (MF), as well as pathways from the Kyoto Encyclopedia of Genes and Genomes (KEGG) (33). To visualize the results, clustering heatmaps were generated using scRNAtoolVis. Pathway inference was executed through PROGENy (34), an R package that leverages a broad collection of publicly available signal perturbation experiments to derive a shared core set of genes responsive to mouse pathways. These data were then integrated with appropriate statistical methods to infer pathway activity based on single-cell transcriptomics.

### 2.6 Comparison of GSEA for lipid-associated macrophage subpopulations

In this study, we employed Gene Set Enrichment Analysis (GSEA) to compare significant gene lists of identified cell subtypes with gene sets filtered from existing literature, aiming to assess homogeneity (35). The GSEA gene sets were curated by filtering Kim et al.’s bulk RNA-seq and scRNA-seq data (18). We applied a p-value threshold of <0.05 and a log2 fold change >1 for bulk transcriptomics, and a p-value <0.05 with log2 fold change >0.5 for single-cell transcriptomics. This process yielded the following gene sets for GSEA analysis: Bulk RNA-seq foamy DOWN-non-foamy UP, Bulk RNA-seq foamy UP non-foamy DOWN, scRNA-seq foamy DOWN non-foamy UP, and scRNA-seq foamy UP non-foamy DOWN. Furthermore, we augmented this gene set by incorporating SPP1+ Mac previously identified in colorectal carcinoma (12). This addition allowed us to compare our identified lipid-associated macrophages with the LAM found in other diseases, as described above, for GSEA comparisons.

### 2.7 Pseudotime and SCENIC analyses

The cell trajectory analysis of macrophage subtypes was conducted using the Monocle2 v2.24.1 package (36). This method maps cells as points in a high-dimensional space that represents their gene expression levels with the aid of inverse graph embedding. After dimensionality reduction, cell trajectories are learned by iteratively adjusting cell positions through a spanning tree while maintaining a reversible mapping between high and low dimensions. The process begins with single-cell RNA sequencing data, constructing the Monocle dataset, setting parameters, estimating size factors and dispersion, screening genes with expression over 0.1, and sorting them through DDRTree dimensionality reduction. Subsequently, cell trajectories were plotted and color-coded according to Seurat clustering, state, and pseudotime. Cell trajectory analysis was further performed on specific genes, revealing changes in their expression patterns across cell states and pseudotime. SCENIC analyses were conducted with reference to the subject databases RcisTarget and GRNboost, using the R packages SCENIC v1.3.1 (37), AUCell v1.18.1, and RcisTarget v1.16.1. The RcisTarget package was employed to analyze excess transcription factor (TF) binding motifs. This involved calculating the Spearman correlation between TFs and potential targets and creating co-expressed genes for each TF via the runGenie3 function. The runSCENIC function generated gene regulatory networks (GRNs), referred to as regulators. Finally, the regulator activity score for each group of regulators in each cell was calculated using the AUCell package.

### 2.8 Identification of LAM subtypes in human heart failure single-cell sequencing

In another single-cell sequencing dataset, specifically derived from a heart failure sample caused by ischemic cardiomyopathy, we applied the same single-cell data processing methods as previously described. Through this analysis, we further investigated the previously identified lipid-associated macrophage (LAM) subtype and GSEA comparisons to confirm the presence of SPP1+ LAM in human. Additionally, we identified Spp1 and Trem2 expressing macrophage subtypes using a single-cell sample obtained from a human heart failure induced by ischemic cardiomyopathy. By performing GSEA, we compared these subtypes with previously constructed gene sets (Bulk RNAseq foamy UP nonfoamy DOWN, scRNAseq foamy UP nonfoamy DOWN, Tumor-specific SPP1+ macrophages) and the Spp1+ subtypes identified in the GSE227088 dataset. This analysis allowed us to compare LAM isoforms with the highly characterized gene set.

### 2.9 Infarct size measurement

To delineate the non-ischemic area (NIA, red) and the area at risk (AAR, white), we employed 2,3,5-triphenyltetrazolium chloride (TTC, Sigma-Aldrich, USA) staining. Initially, cardiac tissues were rapidly frozen for 20 minutes at -80°C. Subsequently, these tissues were thinly sliced into 0.5 mm sections and stained in a 2% TTC solution for 15 minutes in a light-shielded environment. Post-staining, the slices were submerged in 4% paraformaldehyde for an entire night. The extent of the infarct was quantified using Image J software (National Institutes of Health, USA).

### 2.10 Cardiac function measurement

To assess cardiac function in mice, an ultrasound cardiogram (UCG) was conducted. Mice were sedated using 2% isoflurane for the duration of the UCG procedure. We acquired a short-axis view of the left ventricle to evaluate five consecutive cardiac cycles. M-mode tracing facilitated the measurement of the left ventricular end-diastolic dimension (LVEDD), left ventricular end-systolic dimension (LVESD), left ventricular diastolic posterior wall thickness, left ventricular end-diastolic volume (LVEDV), and left ventricular end-systolic volume (LVESV). These parameters were automatically computed by software, based on LVEDD and LVESD values. The left ventricular ejection fraction (LVEF) was determined using the formula: LVEF = 100% × [(LVEDV–LVESV)/LVEDV].

### 2.11 Immunostaining and histology

Hearts were perfused with ice-cold PBS, dissected, embedded in O.C.T. compound (Tissue-Tek #4583), frozen in liquid nitrogen-cold Isopentane (Carl Roth #3927.1) and stored at -80°C. Sections were made with Cryostat (Leica #CM3050 S) at 7µm thickness and stored at -80°C. For immunofluorescence staining, fresh frozen sections were rinsed in PBS (Sigma-Aldrich #P3813-10PAK) for 5 minutes and incubated with Normal Goat Serum Block (Biolegend #927503) for 30 minutes at room temperature. Then, Sections were stained with primary antibodies recognizing F4/80 (Servicebio, GB113373, 1:5000), Spp1 (Servicebio, GB11500, 1:3000), Trem2 (Zenbio, #510482, 1:1000) overnight at 4°C in a humidified chamber. Secondary goat anti-rat Alexa Fluor 594 and LipidTox Deep Red Neutral Lipid Stain (Thermo Fisher) were applied for 1 hour at room temperature. Slides were mounted with ProLong Gold Antifade DAPI (Thermo Fisher).

## 3 Results

### 3.1 Principal cell types in ischemia-reperfusion injury model

**Figure 1:**
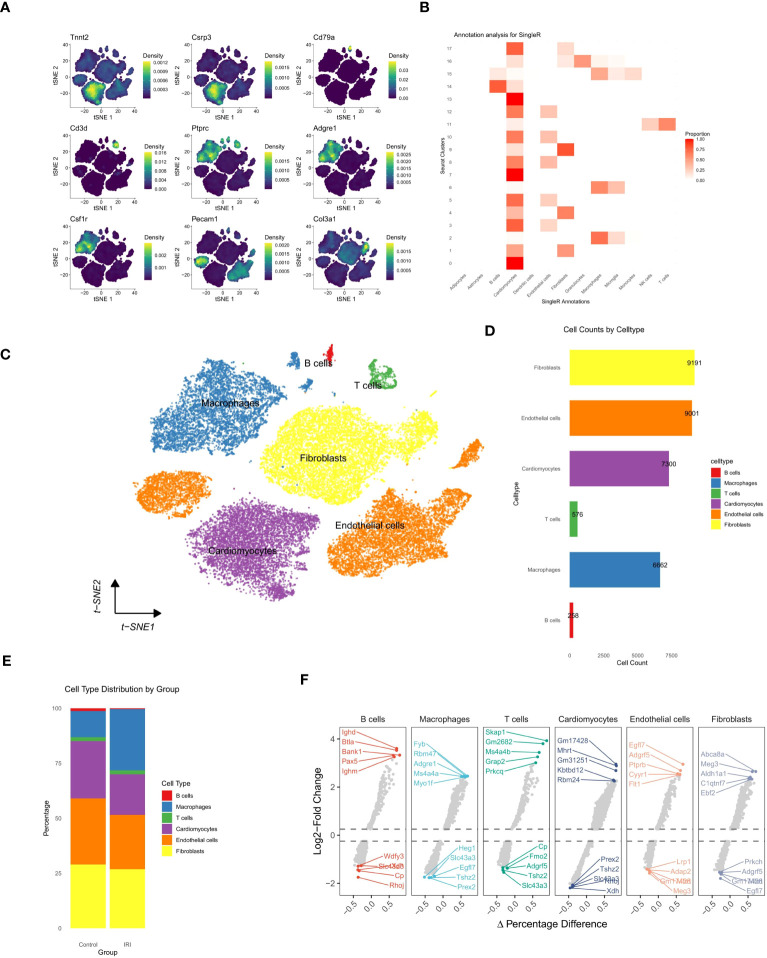
Comprehensive single-cell transcriptomic analysis. **(A)** t-SNE plots displaying the expression density of selected marker genes across identified cell populations. **(B)** Heatmap illustrating the annotation analysis of single cell clusters, the x-axis lists the cell types labeled by SingleR and the y-axis corresponds to the Seurat cluster number. The shade of the color reflects the normalized proportion of each cell type in the cluster, with red indicating a higher proportion. **(C)** t-SNE visualization of cell clusters, color-coded by cell type, with annotations indicating the predominant cell populations. **(D)** Bar graph showing the quantification of cell counts by cell type. **(E)** Stacked bar chart representing the distribution of cell types within control and experimental groups. **(F)** Volcano plots for each cell type detailing gene expression fold change (Log2FC) against the percentage difference in expression, highlighting key genes with significant differential expression (dashed lines indicate thresholds for statistical significance).

Seurat objects were generated based on 50,690 cells and 37,335 gene expression profiles. After quality control (**Supplementary Figure 1**), 20,402 cells and 32,988 gene expression profiles remained for analysis. The unsupervised clustering algorithm in Seurat was applied to categorize these cells into 18 distinct clusters. These clusters were defined based on available biological markers, including cardiomyocytes (Tnnt2+ Csrp3+), B-cells (Cd79a+), T-cells (Cd3d+), macrophages (Ptprc+ Adgre1+ Csf1r+), endothelial cells (ECs) (Pecam1+), and fibroblasts (Col3a1+) (**Figure 1A**). Cell type identification was enhanced by utilizing the SingleR package, which produced annotated heatmaps displaying the normalized proportions of each cell type within the cell population (**Figure 1B**). Detailed high-resolution cell views were generated (**Figure 1C**). Cell counts for various cell types were quantified, and the proportional distribution of cell types between the IRI and control groups was determined (**Figures 1D, E**). Analysis of cell percentages in the IRI and control groups revealed a significant increase in the percentage of macrophages in the IRI group, accompanied by a significant reduction in the percentage of cardiomyocytes. Additionally, there was a moderate decrease in the number of endothelial cells and fibroblasts. Differential gene expression across cell types was also identified (**Figure 1F**, **Supplementary File 1**).

### 3.2 Identifying macrophage subtypes

**Figure 2:**
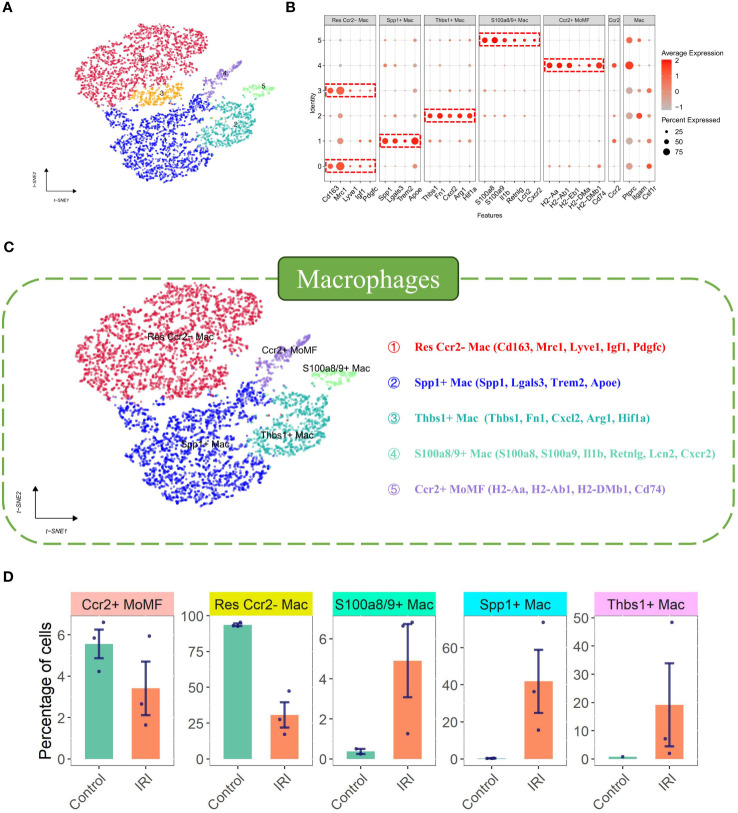
Characterization and comparison of macrophage subpopulations under sham and ischemia-reperfusion injury (IRI) conditions. **(A)** t-SNE plot delineating the distribution of macrophage subclusters, with each color representing a unique subset. **(B)** Dot plot showcasing the feature expression levels across different macrophage subsets, with the size of the dot indicating the percentage of cells expressing the feature and the color intensity corresponding to average expression level. **(C)** Detailed t-SNE plot highlighting five distinct macrophage subsets annotated with key defining markers. **(D)** Comparative bar graphs presenting the percentage of cells within each macrophage subset for sham and IRI conditions, with error bars indicating variability and dots representing individual experimental sample.

**Figure 3:**
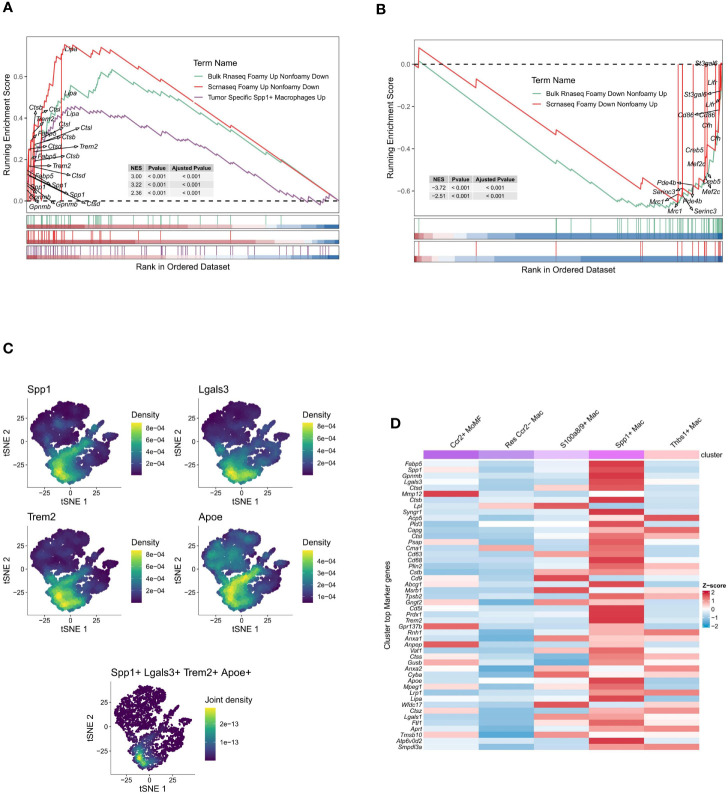
Gene set enrichment and expression profiling in macrophage subpopulations. **(A)** Gene Set Enrichment Analysis (GSEA) plot of up-regulated genes in the foam macrophage population vs. tumor-specific SPP1+ macrophages. The enrichment score (ES) indicates the degree to which a gene set is overrepresented at the top or bottom of a ranked list of genes. **(B)** GSEA plot of up-regulated genes in non-foaming macrophage populations in the same comparison. **(C)** t-SNE plots displaying the density of expression for selected genes (Spp1, Lgals3, Trem2, Apoe) across the macrophage population, with a composite plot showing joint density for a combination of markers. **(D)** Heatmap comparing the z-score normalized expression of gene markers across different macrophage subsets, indicating distinct expression profiles.

Initially, 6662 cells were identified as Macrophages (4768 from the ischemia-reperfusion model group and 1894 from the Sham group). These cells were grouped into 6 Seurat clusters after scaling and normalization (**Figure 2A**). They were annotated based on available biological background (**Figure 2B**) as follows: Res Ccr2- Mac (Resident Ccr2- Macrophage) (Cd163+ Mrc1+ Lyve1+ Ccr2-) (29), MoMF characterized by Ccr2+ and expression of antigen processing/presentation genes (MHC-II genes: H2-Aa, H2-Ab1, H2-Eb1, H2-DMa, H2-DMb1, and Cd74) (29), Thbs1+ Mac (Thbs1+ macrophage) (Thbs1+ Fn1+ Cxcl2+ Hif1a+), and S100a8/9+ Mac (S100a8+ S100a9+ Il1b+ Lcn2+ CXCr2+), considered a distinct inflammatory macrophage subpopulation. Notably, a lipid-associated macrophage (LAM) was identified in myocardial IRI injury, characterized by high expression of Spp1, Trem2, Lgals3, and Apoe (35) (**Figure 2C**), with Trem2 expression being a signature of the LAM (lipid-associated macrophage) subtype (17). The percentages of cells belonging to various macrophage subtypes within the group exhibited a significant increase in the proportions of Thbs1+ Mac, S100a8/9+ Mac, and Spp1+ Mac in the IRI group (**Figure 2D**). Spp1+ Mac exhibited significant positive enrichment of up-regulated genes in foamy macrophages in scRNA-seq (scRNAseq_foamy_UP_nonfoamy_DOWN, NES = 3.22, p adj < 0.001) and bulk RNA sequencing (Bulk_RNAseq_foamy_UP_nonfoamy_DOWN, NES = 3.00, p adj < 0.001) datasets, as well as in tumor-specific SPP1+ macrophages (Tumor_specific_SPP1+_macrophages_UP, NES = 2.36, p adj < 0.001) (**Figure 3A**). Similarly, Spp1+ Mac showed negative enrichment of genes upregulated in non-foamy macrophage populations (scRNAseq_foamy_DOWN_nonfoamy_UP, NES = -2.51, p adj < 0.001, and Bulk_RNAseq_foamy_DOWN_nonfoamy_UP, NES = - 3.72, p adj < 0.001) (**Figure 3B**, **Supplementary File 2**). The expression distribution and density status of characteristic genes Spp1, Trem2, Lgals3, and Apoe were assessed (**Figure 3C**). Furthermore, when evaluating the relative expression of the top 50 genes in the scRNAseq_foamy_UP_nonfoamy_DOWN dataset defined by Kim et al. compared to all other macrophage subtypes, Spp1+ Mac showed the greatest enrichment (**Figure 3D**). The high degree of concordance observed between the differentially expressed genes defining Spp1+ Mac and the known foamy macrophage dataset validates the characterization of these cells as LAM. Based on these results, we identified this cell cluster as the SPP1+ Lipid-Associated Macrophage (LAM) subtype, a previously unseen cluster in MIRI.

### 3.3 Enrichment and pathway analysis results of macrophage subtypes

**Figure 4:**
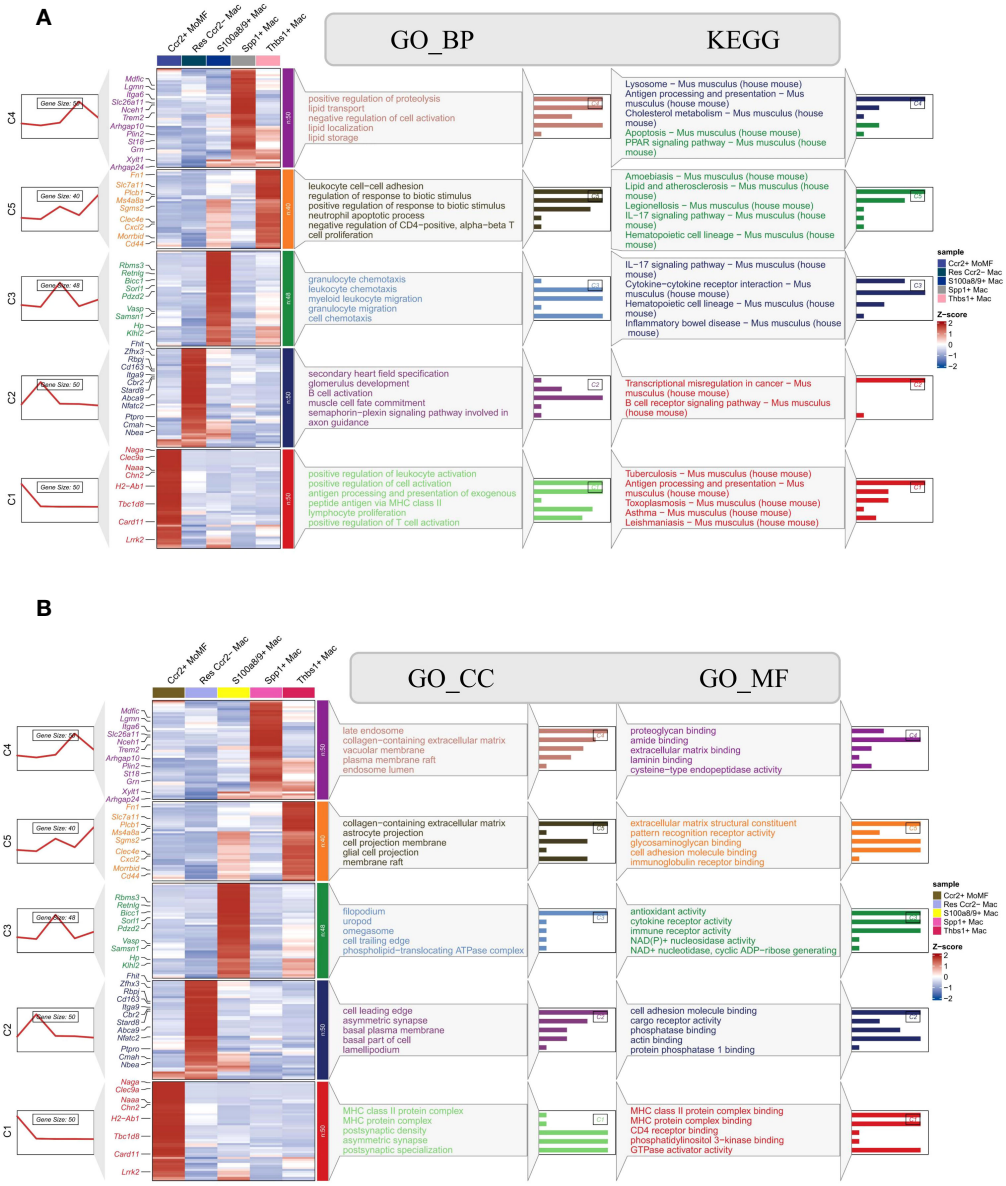
Functional annotation and pathway enrichment analysis in macrophage subsets. **(A)** Heatmap and enrichment pathways for Gene Ontology Biological Process (GO_BP) categories and Kyoto Encyclopedia of Genes and Genomes (KEGG) pathways across different macrophage subsets, with annotations indicating significant biological processes and pathways enriched in each subset. **(B)** Corresponding heatmap and enrichment analysis for Gene Ontology Cellular Component (GO_CC) and Molecular Function (GO_MF), illustrating the subcellular localization and functional attributes of the gene products. Each column represents a macrophage subset, and rows correspond to specific GO terms or KEGG pathways, with color coding indicating the Z-score normalized level of gene expression. The sidebars connect specific genes to their associated biological processes, cellular components, and molecular functions, as well as to their respective KEGG pathways, highlighting the diverse functional roles of these subsets in various biological contexts.

Enrichment analyses revealed distinct cellular functions among different macrophage subtypes in the context of MIRI, with particular emphasis on the SPP1+ LAM subtype, which exhibited significant enrichment across various biological processes (BP), cellular components (CC), and molecular functions (MF). In terms of biological processes, this subtype was associated with critical processes, including the positive regulation of protein degradation, lipid transport, negative regulation of cellular activation, lipid localization, and lipid storage. Regarding cellular components, this subtype was primarily localized in key cellular areas such as late endosomes, collagen-containing extracellular matrices, vesicle membranes, plasma membrane microdomains, and endosomal lumens. At the molecular function level, it encompassed essential functions such as proteoglycan binding, amide binding, extracellular matrix binding, laminin binding, and cysteine endonuclease activity. Furthermore, this subtype displayed significant enrichment in several pathways, including pivotal pathways like the Lysosome pathway, antigen processing and presentation pathway, cholesterol metabolism pathway, apoptosis pathway, and PPAR signaling pathway (**Figures 4A, B**, **Supplementary File 3**). Progeny pathway analysis revealed a notable downregulation of the MAPK signaling pathway in SPP1+ LAMs compared to other macrophage subsets in the context of murine ischemic reperfusion injury (**Supplementary Figure 2A**) and human heart failure (**Supplementary Figure 2B**). This subdued MAPK activity may delineate a specific pathophysiological trajectory in SPP1+ LAMs during cardiac distress and disease progression.

### 3.4 Regulon network analysis with SCENIC

**Figure 5:**
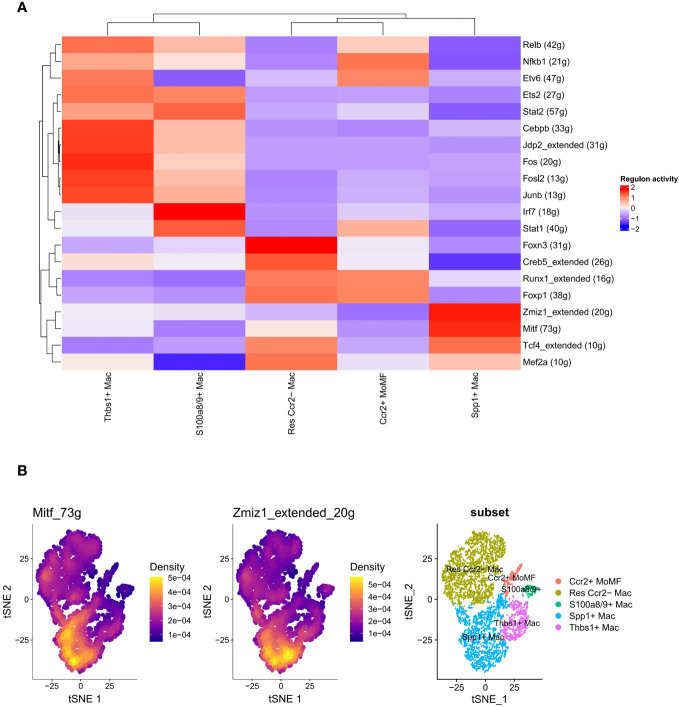
Transcription factor activity and gene expression heterogeneity in macrophage subsets. **(A)** Heatmap depicting the inferred activity of transcription factors across different macrophage subsets, with colors representing the regulon activity score (red for higher activity and blue for lower activity). Each row corresponds to a transcription factor, with the number of target genes indicated in parentheses. Hierarchical clustering on the top and left reflects the similarity in transcription factor activity patterns among subsets and transcription factors, respectively. **(B)** t-SNE plots highlighting the expression density of genes Mitf_73g and Zmiz1_extended_20g in the macrophage population, and an annotated t-SNE plot showing the distribution of macrophage subsets, each color-coded for a specific subset. The density color scale indicates the level of gene expression within the cell population.

Prior research has demonstrated the pivotal role of transcription factors, such as Irf1, Egr1, and Stat1, in the regulation of macrophage polarization (38, 39). We further explored the correlation between the activities of these regulators (transcription factors and their target genes) and the differentiation of macrophage subtypes using SCENIC analysis. A total of 44 regulators exhibiting high activity levels were identified, and a clustered heatmap was generated, focusing on the top 20 regulators (**Figure 5A**). Notably, Mitf (73g) and Zmiz1_extended (20g) exhibited specific regulatory roles in the SPP1+ LAM subtype (**Figure 5B**).

### 3.5 Pseudotime analysis results

**Figure 6:**
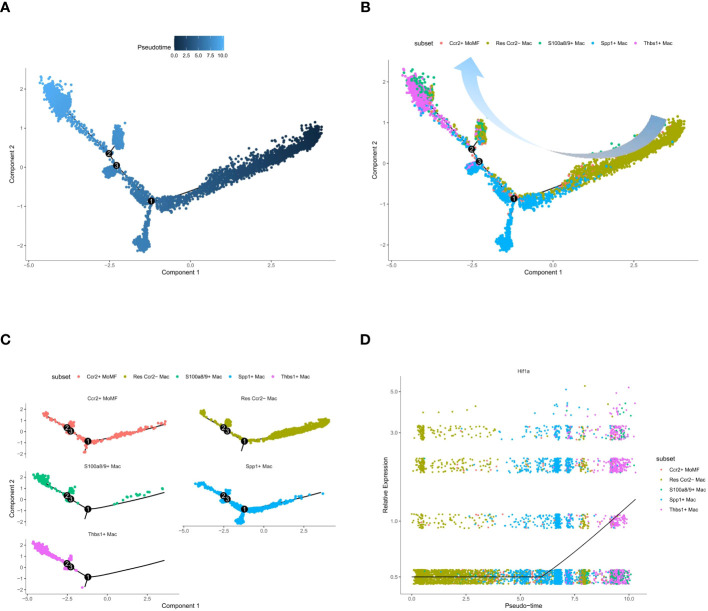
Pseudotime trajectory analysis of macrophage subsets and gene expression dynamics. **(A)** Monocle pseudotime trajectory plot, where each point represents a single cell. **(B)** Trajectory plot with cells color-coded by macrophage subset, indicating the branching structure and distribution of subsets along the trajectory. **(C)** Separate trajectory plots for each macrophage subset. **(D)** Scatter plot of gene expression level against pseudotime for Hlf1a, demonstrating the dynamic regulation of gene expression along the developmental continuum. The black line indicates the trend of gene expression change over pseudotime.

Pseudotime trajectory analysis of macrophage subtypes revealed that Thbs1+ Mac and S100a8/9+ Mac were the last clusters to differentiate (**Figures 6A, B**). Enrichment analysis showed that both Thbs1+ Mac and S100a8/9+ Mac activated the IL-17 signaling pathway. Interleukin 17 (IL-17) is a highly versatile pro-inflammatory cytokine, and its signaling pathway is critical for various processes, including host defense, tissue repair, and the pathogenesis of inflammatory diseases (40). It also plays a significant role in immune cell chemotaxis, migration, and adhesion, with Thbs1+ Mac and S100a8/9+ Mac identified as pro-inflammatory macrophage clusters. Other macrophage subtypes were consistently present throughout the pseudotime analysis and persisted throughout the ischemia-reperfusion process (**Figure 6C**). We also obtained LAM pseudotime traces. Notably, hypoxia-inducible factor-1a (Hif1a) is a transcription factor that governs various cellular responses to hypoxia (41). We found that LAM differentiation was associated with an upregulation of Hif1a expression (**Figure 6D**) and an enhanced hypoxic environment. Therefore, it is reasonable to assume that LAM differentiation positively correlates with the extent of the hypoxic conditions. Pseudotime trajectories of regulators, identified through SCENIC, and highly characterized LAM genes were plotted. It was observed that the peak expression of all genes occurred in the SPP1+ LAM cluster (**Supplementary Figure 3**).

### 3.6 Validation of human ischemic cardiomyopathy single-cell data set

**Figure 7:**
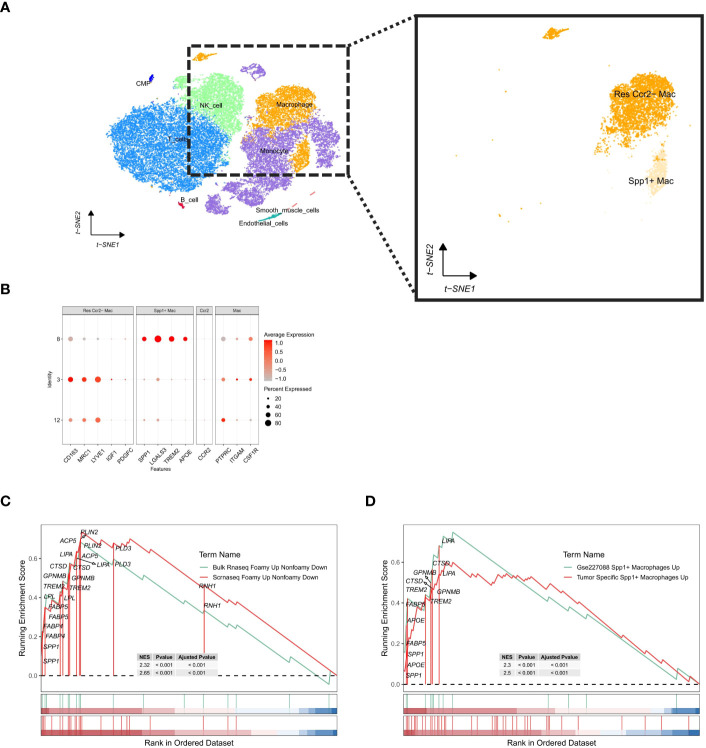
Single-cell analysis of human ischemic cardiomyopathy cell subpopulations and identification of Spp1+ LAM subtypes. **(A)** t-SNE visualization of cell populations. Enlarged view of macrophage subpopulation further distinguishes resident Ccr2- macrophages from Spp1+ macrophages. **(B)** Characterization plot showing expression levels of a range of genes in macrophage subpopulations, with dot size indicating the percentage of cells expressing a particular gene and color intensity reflecting the average level of expression. **(C)** Gene Set Enrichment Analysis (GSEA) plot of genes up-regulated in foamy macrophages. **(D)** GSEA plot of upregulated genes in GSE27088 Spp1+ macrophages and tumor-specific SPP1+ macrophages. The enrichment score (ES) indicates the degree to which a gene set is overrepresented at the top or bottom of a ranked list of genes.

In human samples afflicted with ischemic cardiomyopathy-induced heart failure, a single-cell landscape of macrophages was constructed using the same approach (**Figure 7A**). The distribution of cells from the single-cell data sample and the results of routine analyses are detailed in the accompanying (**Supplementary Figure 4**). Subsequently, we identified a macrophage subtype co-expressing Spp1 and Trem2 (**Figure 7B**). Comparative analyses were performed using the Gene Set Enrichment Analysis (GSEA) method, demonstrating a significant positive enrichment of upregulated genes in foam macrophages in both the single-cell RNA-seq dataset (scRNAseq_foamy_UP_nonfoamy_DOWN, NES = 2.65, p adj < 0.001) and bulk RNA-seq data (Bulk_RNAseq_foamy_UP_nonfoamy_DOWN, NES = 2.32, p adj < 0.001) relative to the previously constructed gene sets (**Figure 7C**). This enrichment was also observed in tumor-specific SPP1+ macrophages (Tumor_specific_SPP1+_macrophages_UP, NES = 2.5, p adj < 0.001) as well as in the Spp1+ LAM subset identified in the GSE227088 dataset (GSE227088 Spp1_LAM_UP, NES = 2.2, p adj < 0.001) (**Figure 7D**, **Supplementary File 4**). Notably, the highly consistent overlap between the differentially expressed genes in human-identified LAM and mouse-identified Spp1+ LAM further confirms the presence of SPP1+ LAM in human samples, underscoring their highly conserved nature as LAM cells.

### 3.7 Co-staining of LAM markers

**Figure 8:**
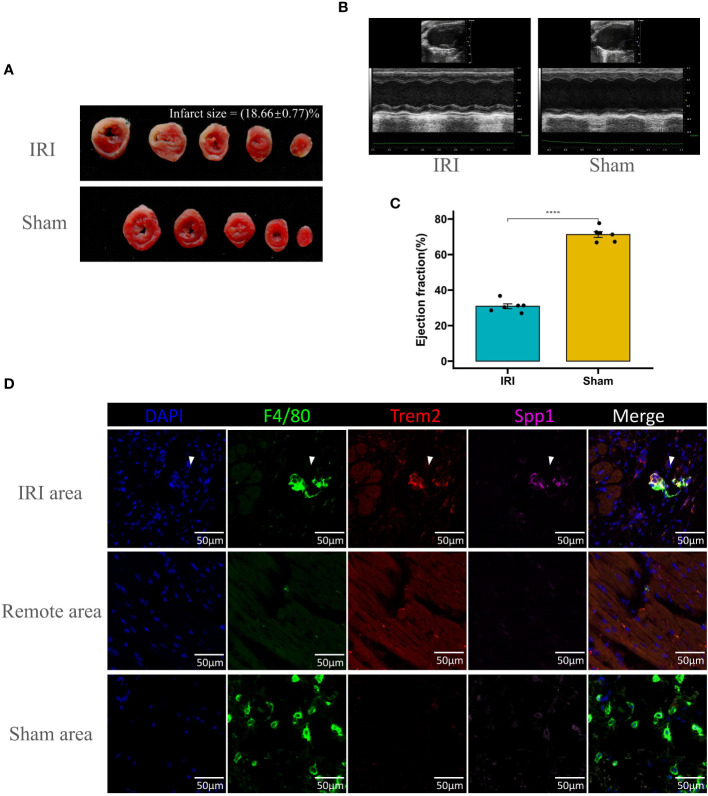
Evaluation of ischemia-reperfusion injury (IRI) and Co-Staining of LAM Markers in cardiac tissue. **(A)** Representative images of TTC-stained heart sections from IRI and sham-operated mice, showing the extent of infarction (white areas) with quantification of infarct size expressed as a percentage of the total heart area. **(B)** Echocardiographic analysis of left ventricular function in IRI and sham mice, with M-mode images displaying ventricular dimensions and wall motion. **(C)** Bar graph comparing the ejection fraction percentages between IRI and sham groups, indicating a significant decrease in cardiac function post-IRI (****p < 0.0001). **(D)** Immunofluorescence staining for macrophage markers (F4/80, green; Trem2, red; Spp1, magenta) in the IRI affected area, remote area, and sham-operated heart tissue. DAPI (blue) is used to stain nuclei. The merged images show colocalization of the markers within the macrophages. Scale bars: 50μm.

Infarct size was significantly increased in IRI models, as demonstrated by the distinct pale staining in TTC-stained heart sections, indicative of tissue death, compared to the uniform red staining observed in sham-operated controls, quantitatively representing 18.66 ± 0.77% of the total heart area (**Figure 8A**). Cardiac function, assessed through echocardiographic measurements, was notably compromised in IRI subjects, with M-mode imaging revealing substantial impairments in ventricular wall motion and dimensions (**Figure 8B**). This functional deficit was further substantiated by a statistically significant reduction in the ejection fraction, with IRI subjects exhibiting a marked decrease in comparison to sham subjects (∗∗∗∗P<0.0001) (**Figure 8C**). F4/80 is commonly utilized as a universal marker for macrophages, whereas Trem2 expression is a key characteristic of Lipid-Associated Macrophages (LAM). Remarkably, macrophages co-expressing Spp1 and Trem2 were identified within the infarct zone of the mouse myocardial ischemia-reperfusion model (**Figure 8**). Conversely, no macrophages co-expressing Spp1 and Trem2 were detected in the remote zone or in the Sham group. These experiments provide conclusive evidence of the presence of LAM.

## 4 Discussion

The pathogenesis of cardiovascular disease is intricate, and thus, targeting inflammation alone with nonspecific approaches may not suffice to significantly enhance clinical outcomes (42, 43). Consequently, more precise intervention strategies are required, which encompass interventions targeting specific sites and cellular subpopulations. One alternative approach involves the activation of specific immune cell subpopulations to induce a protective inflammatory response. Leveraging single-cell RNA-seq data from myocardial ischemia-reperfusion injury (MIRI) in combination with existing biological knowledge, we constructed a single-cell atlas of macrophages involved in MIRI. In doing so, we successfully identified a subpopulation of macrophages characterized by high expression of Spp1 and Trem2 genes, referred to as SPP1+ Trem2 + macrophages with a tissue-acquired Lipid-Associated Macrophage (LAM) gene expression signature. Trem2, a primary macrophage sensor of extracellular lipids, plays a pivotal role in eliciting conserved and protective immune responses in the context of metabolic homeostasis disruption (10). Its downstream signals serve as danger signals indicating tissue injury or disease, while also contributing to tissue development and maintenance within specific microenvironments (10, 44). Trem2 significantly regulates cellular function by promoting phagocytosis, dampening inflammation, and enhancing macrophage survival (45, 46). On the other hand, Spp1, also known as osteoblastin, is an extracellular matrix protein and proinflammatory cytokine implicated in numerous physiological and pathophysiological processes (47). The existence of the LAM subpopulation has been widely documented in various diseases, including murine mammary tumors (35), human atherosclerosis (10), fatty liver (48), colorectal liver metastases (49), and more. These studies suggest that this macrophage state exhibits a degree of conservation across different species and disease contexts.

Furthermore, it’s noteworthy that not only macrophages but also microglia associated with Trem2-dependent diseases exhibit characteristics of LAM (19). This observation suggests that the development of this state is not solely contingent on the cell’s origin but is significantly influenced by the local tissue microenvironment. Research has indicated that phagocytosis may be diminished in lipid-rich macrophages (35). Conversely, in SPP1-expressing macrophages, integrins have been proposed to enhance phagocytosis (50), with the underlying mechanisms intricately tied to environmental factors. The presence of the LAM state has been observed in bile duct macrophages (48), and LAM features have been induced by lipids during monocyte-to-macrophage differentiation *in vitro*, implying that sustained exposure to elevated lipid levels may drive the acquisition of LAM features (11). It’s worth noting that the acquisition of LAM features isn’t exclusively governed by lipid-related pathways; other microenvironmental factors play a significant role. Exposure to calcium, a damage-associated molecular pattern released by necrotic cells, robustly induces the expression of characteristic LAM markers in human monocyte-derived macrophages. Studies, such as that of Murthy et al. (51), have demonstrated that human monocytes exposed to calcium (released by necrotic cells to activate macrophages) (52) differentiate into macrophages with elevated SPP1 expression, a characteristic feature of Trem2 + LAM. This may also elucidate the observed high levels of SPP1 expression in LAM macrophages. SPP1, also known as osteopontin, induces potent pro-inflammatory activation of monocytes, further aggravating inflammation (51, 53).

Within the realm of lipid metabolism, the SPP1+ LAM subtype unveils its remarkable regulatory role. In biological processes, it is intricately involved in lipid transport, localization, and storage. Moreover, at the molecular-functional level, this isoform exhibits specific functions such as extracellular matrix binding and laminin binding. Furthermore, KEGG enrichment analysis reveals that pathways associated with lipid metabolism, including cholesterol metabolism and the peroxisome proliferator-activated receptor (PPAR) signaling pathway, are significantly enriched in the SPP1+ LAM isoform. This suggests that this isoform plays a pivotal role in modulating lipid metabolic pathways and associated signaling. SPP1+ LAM isoforms assume a dual role in the domain of lipid metabolism. On one hand, they play a crucial role in supporting the homeostasis and regulation of intracellular lipid metabolism by participating in lipid transport, localization, storage, and molecular interactions with lipids, as well as regulating pathways linked to lipid metabolism (54). On the other hand, the transcriptional profile of LAM may be induced by pathological lipid loading or tissue damage, leading to heightened inflammatory effects in the overall process of cardiac healing. The accumulation of LAM in the infarcted area (17), phagocytosis of deceased cells, and exposure to damage-associated molecular patterns released by these cells may be associated with the acquisition of the SPP1+LAM profile by macrophages.

The results of PROGENy analysis revealed that SPP1+ LAM is distinguished by the down-regulation of the mitogen-activated protein kinase (MAPK) signaling pathway in comparison to other macrophage subtypes. It is well-established that the p38 MAPK signaling cascade plays a crucial role in cardiac remodeling, and inhibiting the MAPK pathway has been shown to mitigate the inflammatory response and myocardial remodeling in myocardial infarction mice (55, 56). This finding provides a significant pathway insight, suggesting that SPP1+ LAM may influence cardiac remodeling by modulating the MAPK pathway. Additionally, SCENIC Regulon network analysis identified Mitf (73g) and Zmiz1_extended (20g) as regulators exhibiting high regulatory activity in LAM. It has been proposed that Mitf regulates Gpnmb expression in macrophages through direct binding and transactivation of the Gpnmb promoter (57). Gpnmb serves as a reliable LAM marker. These findings suggest potential pathways governing the differentiation of macrophage subtypes, which will require further evaluation in a suitable model system. The precise roles of transcription factors and their contributions to the pathogenesis of MIRI are subjects for future investigations. Meanwhile, targeting the anti-inflammatory effects of Trem2 may present a novel therapeutic strategy for MIRI treatment (58). Conventional approaches involve targeting signaling pathways that regulate Trem2 expression and activation to achieve the beneficial effect of modulating Trem2 macrophage function. In a recent study, Jung et al. (59) introduced a novel approach involving the administration of soluble TREM2 + macrophages. This study observed an increase in soluble Trem2 (sTrem2) expression beginning on day 3 after myocardial infarction, peaking on day 7. They administered sTrem2 mixed with gelatin hydrogel (sTrem2-GH) *in vivo* and demonstrated that sTrem2 promotes functional and structural improvement in the injured mouse heart. This improvement was evidenced by a reduction in myocardial infarct size and enhancements in echocardiographic left ventricular systolic function 28 days after myocardial infarction.

Several limitations persist in this study. First, although our study encompasses a considerable number of samples, we acknowledge that the sample size may potentially influence the generalizability of our findings. However, we believe that our research is robust and reliable within the context of the sample size involved. Secondly, while we successfully identified SPP1+ LAM in the context of ischemia-reperfusion injury (MIRI) using single-cell datasets from both mice and humans, and confirmed its conservation across species, our mechanistic investigations primarily relied on bioinformatics methods and comprehensive literature analysis. Consequently, further experimental studies are warranted to delve deeper into its mechanistic actions. Furthermore, despite pinpointing SPP1+ LAM as a potential regulator of cardiac remodeling, additional research is essential to unravel its systemic properties. This involves elucidating the origins of SPP1+ LAM, assessing the impact of extracellular environmental factors, and elucidating the intricate intracellular signaling cascades that underlie their regenerative effects in the infarcted heart. Such investigations will contribute to a more comprehensive understanding of the role of LAM in disease progression and aid in tailoring therapeutic strategies to specific disease populations.

## 5 Conclusion

Single-cell RNA-seq analysis of MIRI mouse hearts revealed significant macrophage heterogeneity, unveiling a novel subpopulation characterized by enrichment in lipid metabolism, extracellular matrix remodeling, and the regulation of cell signaling in this context, which we termed SPP1+ LAM. This study also validated in human heart failure samples the presence of the LAM subtype, a highly conserved macrophage subtype that plays a dual role in different pathological conditions, with both beneficial and detrimental effects. Exploring the utilization and regulation of these cells may open up a new avenue of research aimed at mitigating ischemia-reperfusion injury.

## Data availability statement

The datasets presented in this study can be found in online repositories. The names of the repository/repositories and accession number(s) can be found in the article/**Supplementary Material**.

## Ethics statement

The animal study was approved by The Animal Experimentation Ethics Committee of the First Affiliated Hospital of Nanchang University (Approval Code: CDYFY-IACUC-202209QR009). The study was conducted in accordance with the local legislation and institutional requirements.

## Author contributions

YJ: Writing – original draft. WY: Writing – original draft. TH: Writing – original draft. HP: Writing – original draft. FH: Writing – original draft. YY: Writing – original draft. XL: Writing – original draft. SL: Writing – original draft. JZ: Writing – review & editing. XD: Writing – review & editing.
