# Supplementary material for: Unveiling macrophage diversity in myocardial ischemia-reperfusion injury: identification of a distinct lipid-associated macrophage subset
Source: Front Immunol. 2024 Feb 21;15:1335333. doi: 10.3389/fimmu.2024.1335333 (PMC10915075; doi:10.3389/fimmu.2024.1335333)
Supplement: Supplementary file 5 [file DataSheet_1.docx]

Supplementary Material

Unveiling Macrophage Diversity in Myocardial Ischemia-Reperfusion Injury: Identification of a Distinct Lipid-Associated Macrophage (LAM) Subset

Ying Jiang^1^†, Wenpeng Yu^1^, Tie Hu^1^, Huaxi Zou^1^, Xufeng Liu^4^, Songqing Lai^3^, Xiao Dong^1^*, Jianliang Zhou^2^*

^1^ Department of Cardiovascular Surgery, The Second Affiliated Hospital of Nanchang University, Nanchang, China

^2^ Department of Cardiovascular Surgery, Zhongnan Hospital of Wuhan University, Wuhan, China

^3^Department of Cardiovascular Surgery, The First Affiliated Hospital of Nanchang University, Nanchang, China

^4^ Department of Haematology, Ganzhou People's Hospital, Ganzhou, China

**Correspondence:**

Xiao Dong

Dongshi22000@163.com

Jianliang Zhou
[zhoujianliang2010@163.com](mailto:zhoujianliang2010@163.com)

***First authorship:** These authors share first authorship

# Supplementary Materials and Methods

# Supplementary Figures and Files


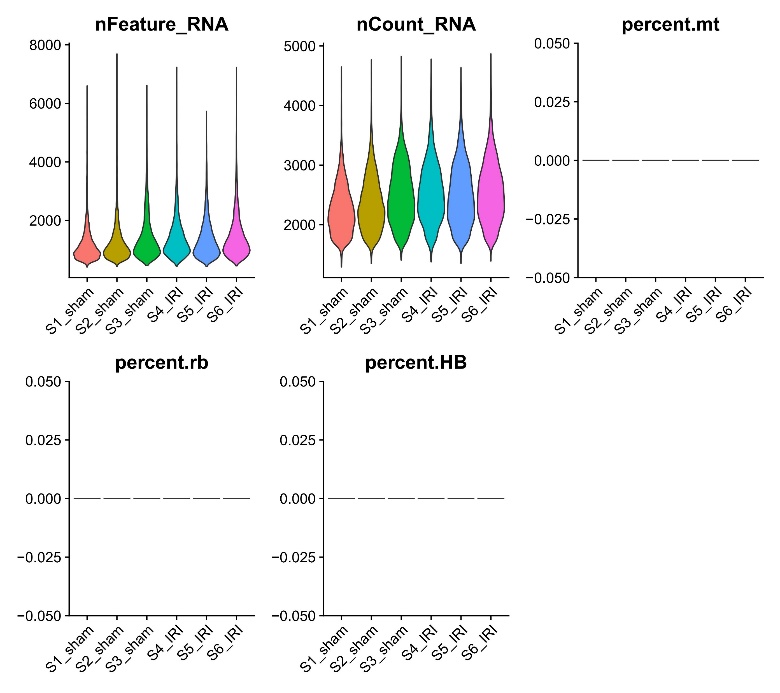

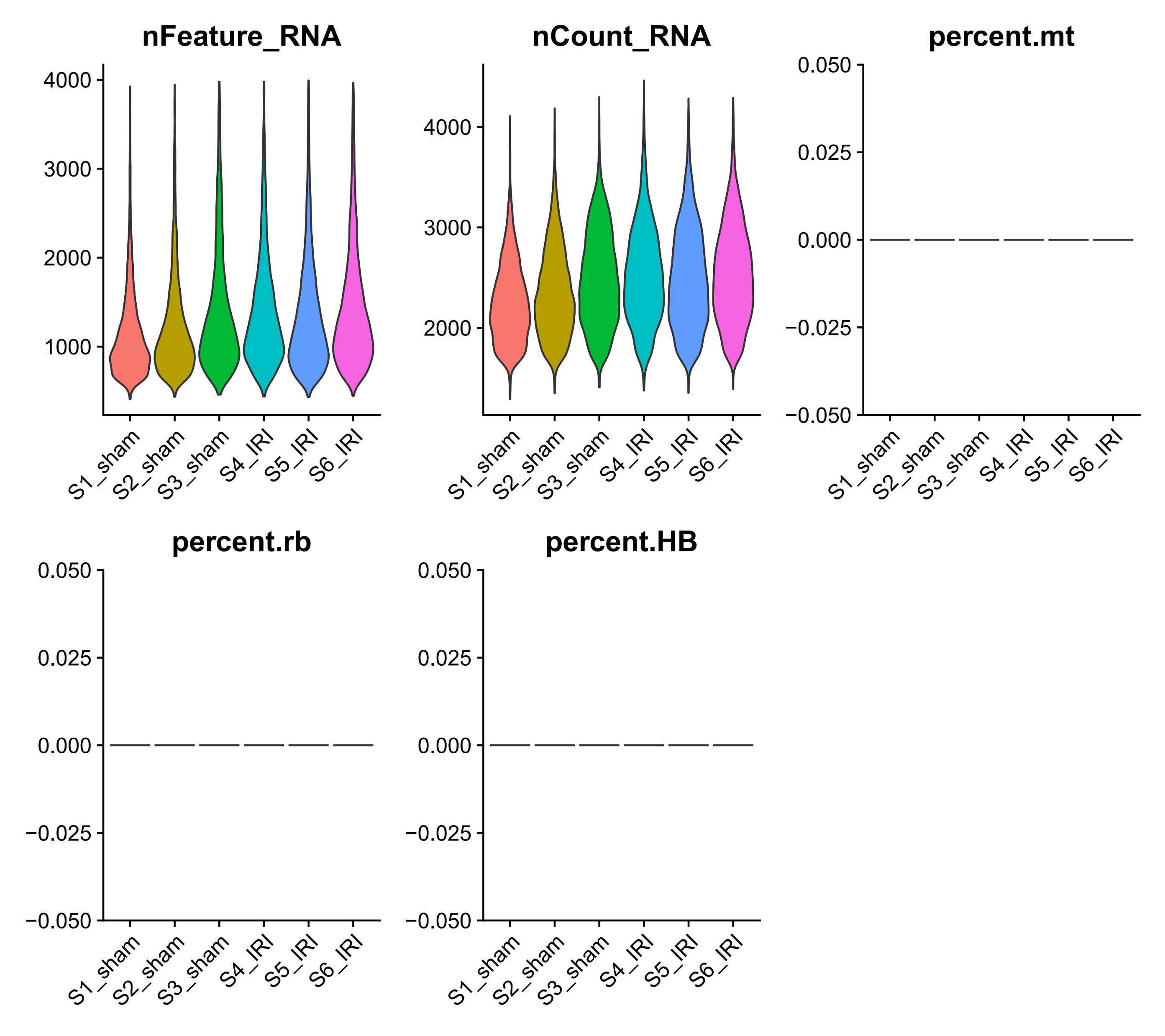


pre-quality control

Post-quality control

Supplementary Figure 1. Comparison of Pre- and Post-Quality Control Filtering for Eliminating Unqualified Cells and Genes.


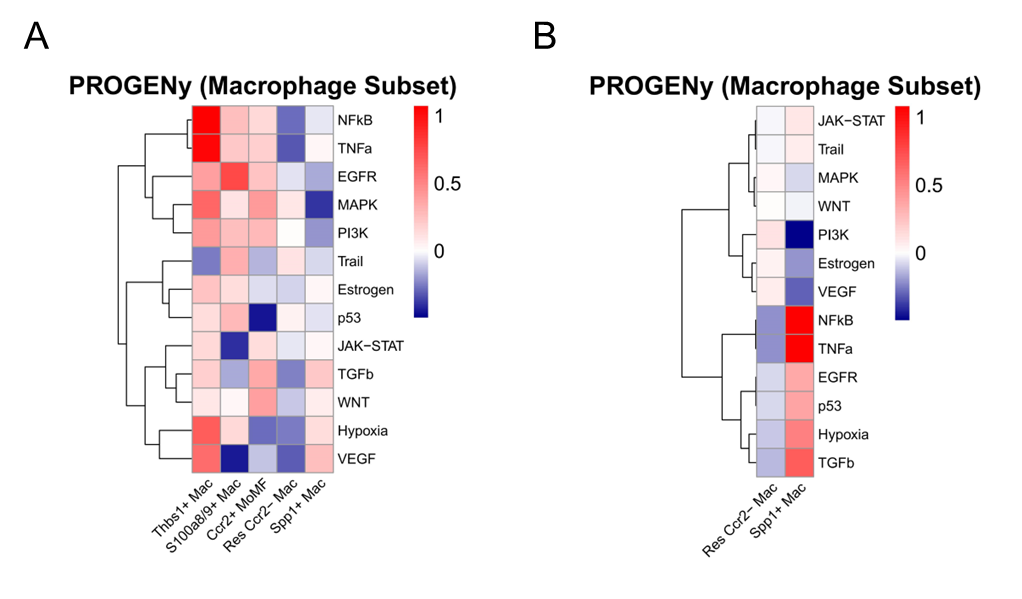


Supplementary Figure 2. MAPK Pathway Modulation in Spp1+ Macrophages. The heatmap analysis indicates a downregulated MAPK pathway in Spp1+ macrophages from both ischemic reperfusion injury (A) and heart failure (B) models, suggesting a potential suppression or regulatory mechanism of MAPK signaling in these pathological states.


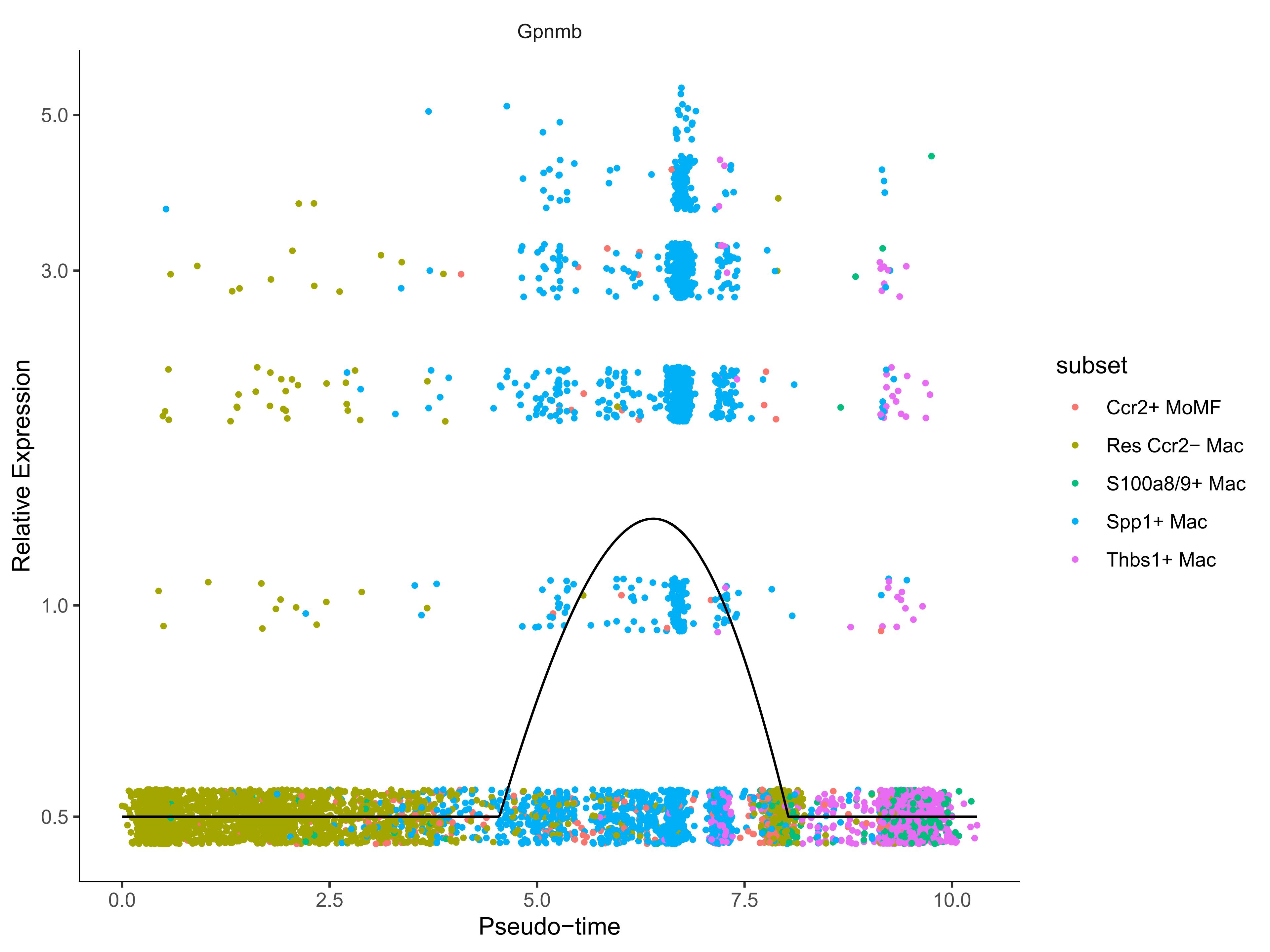

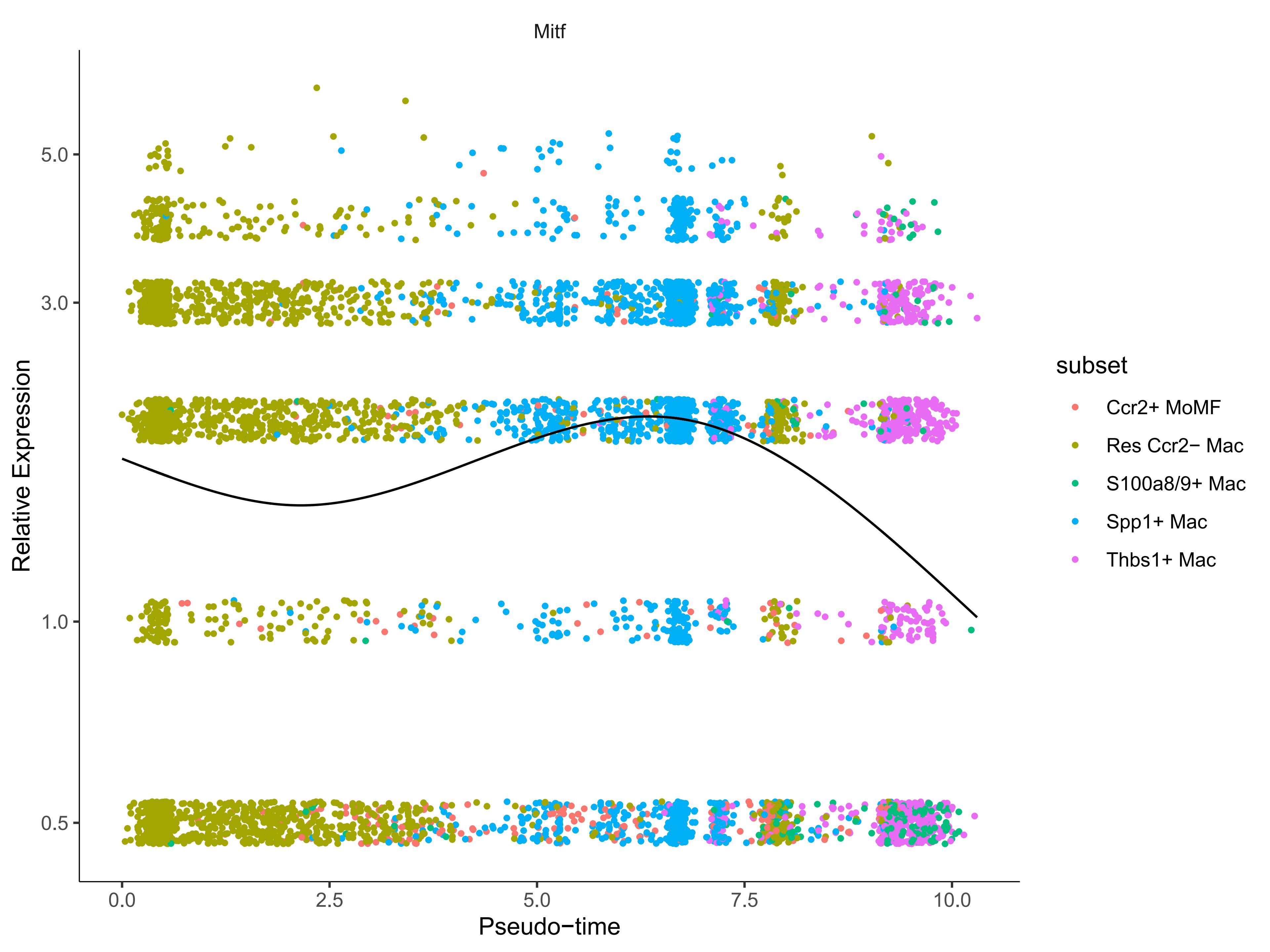

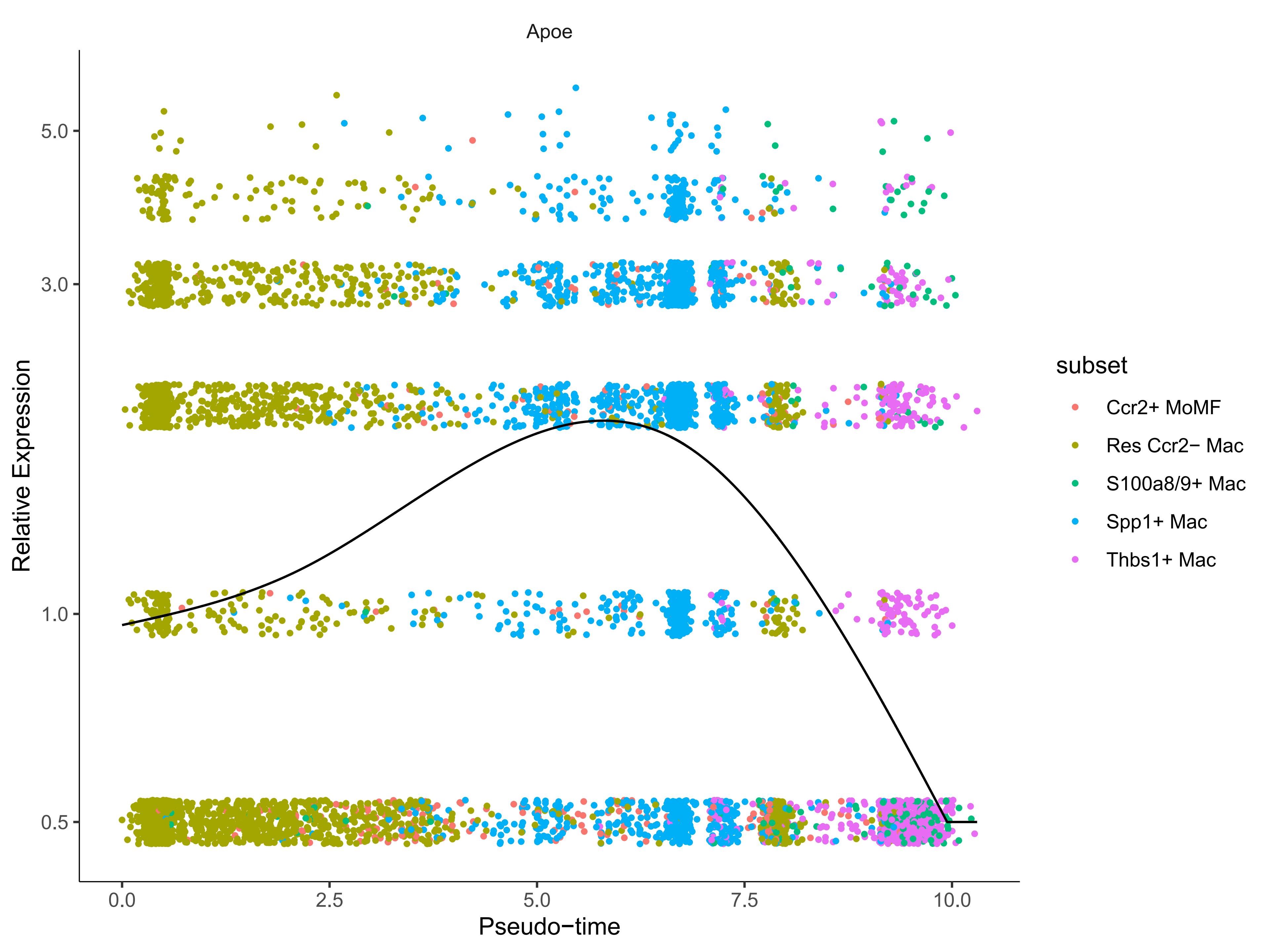

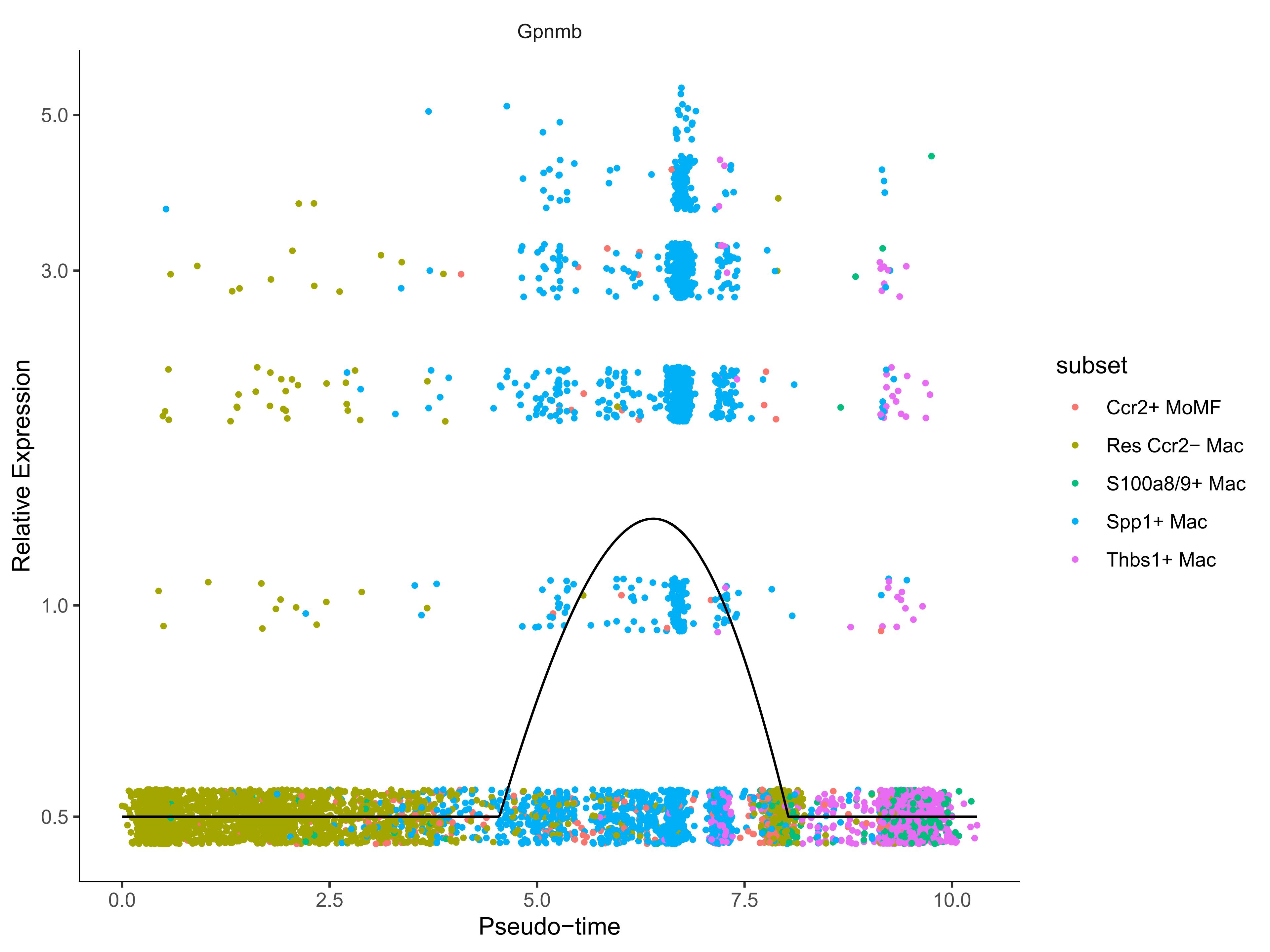

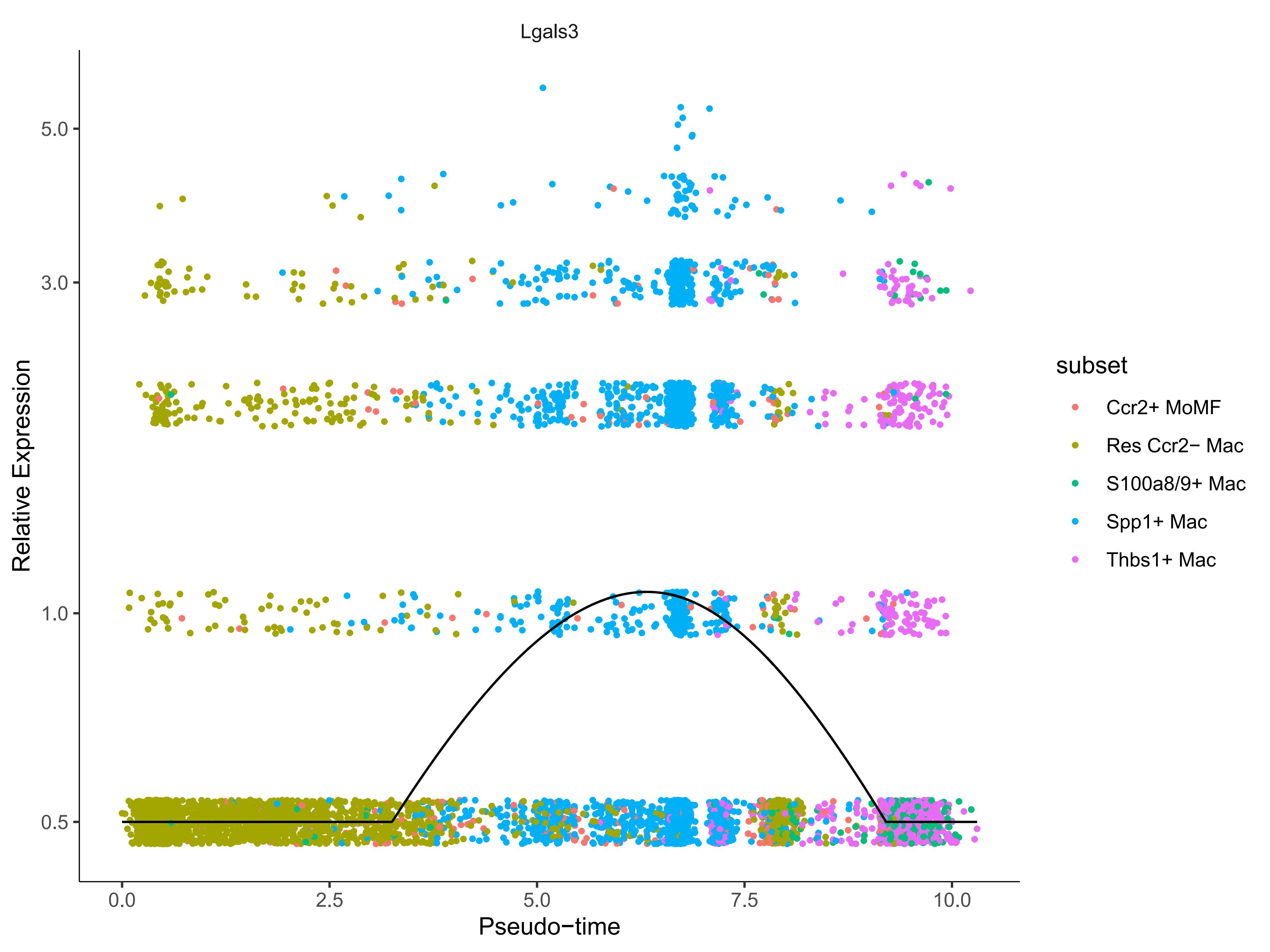

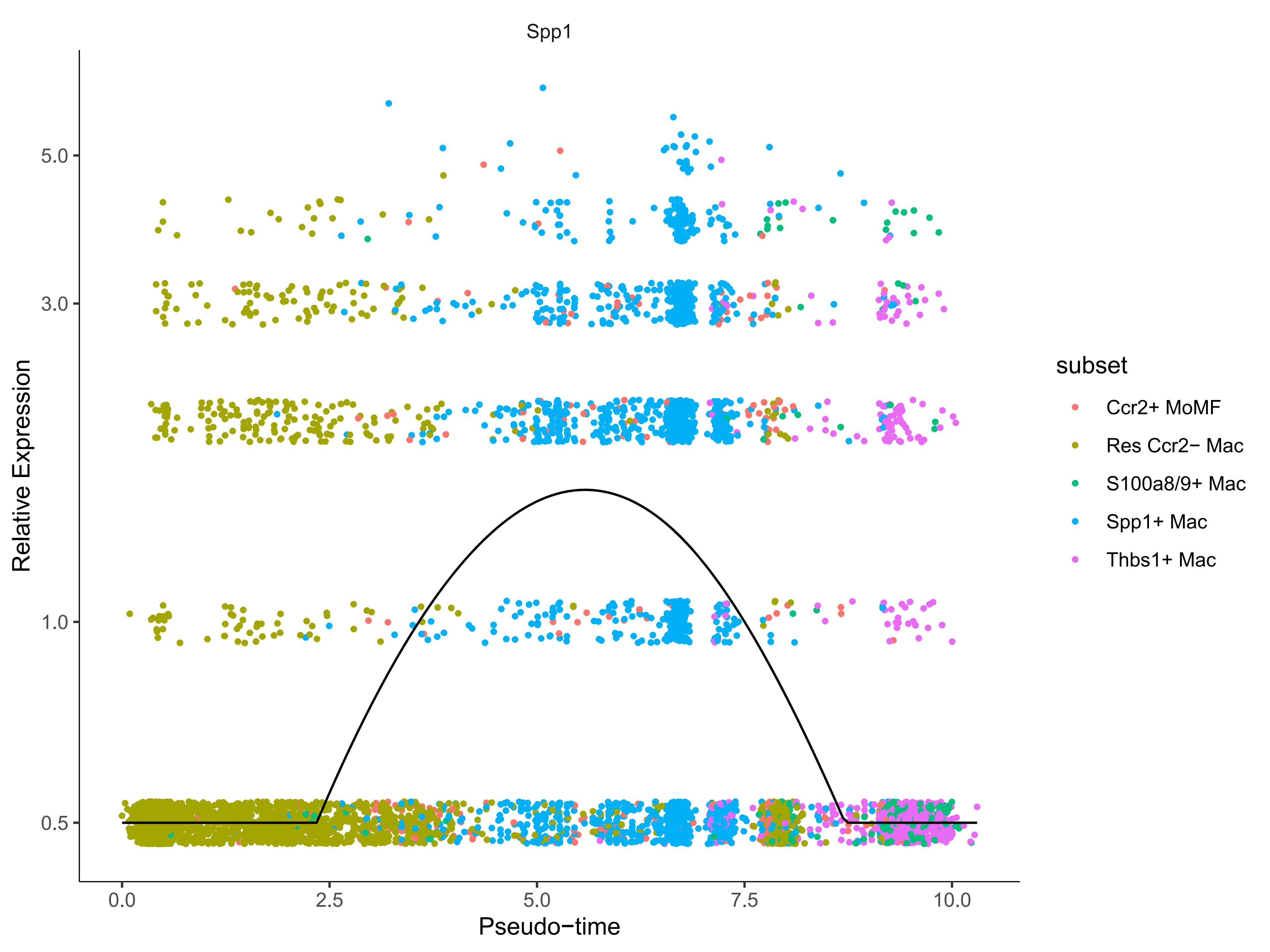

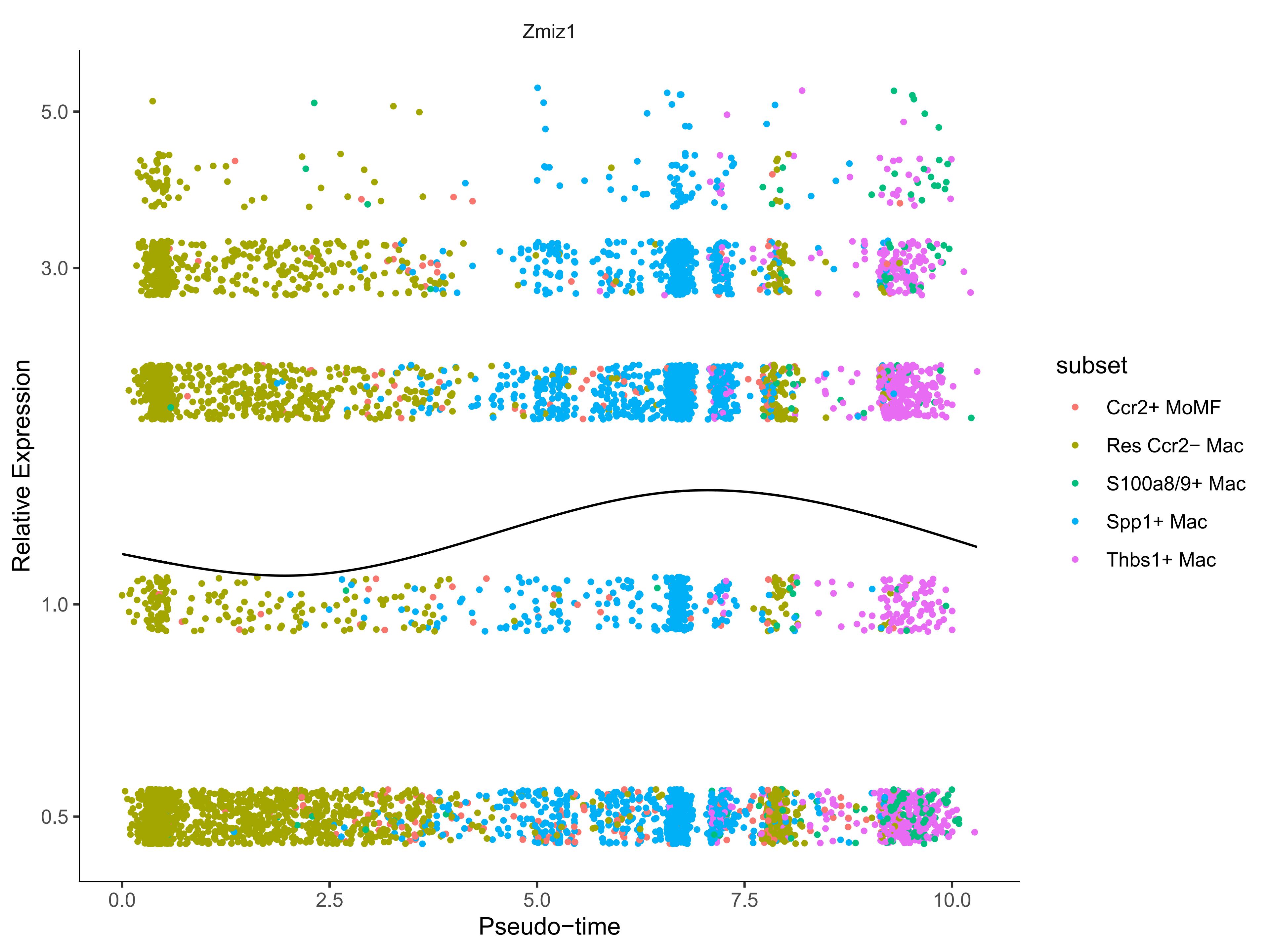


Supplementary Figure 3. Pseudotime Trajectories of SCENIC-Identified Regulators and Highly Characterized LAM Genes Showing Peak Expression in the Macrophage subtype.


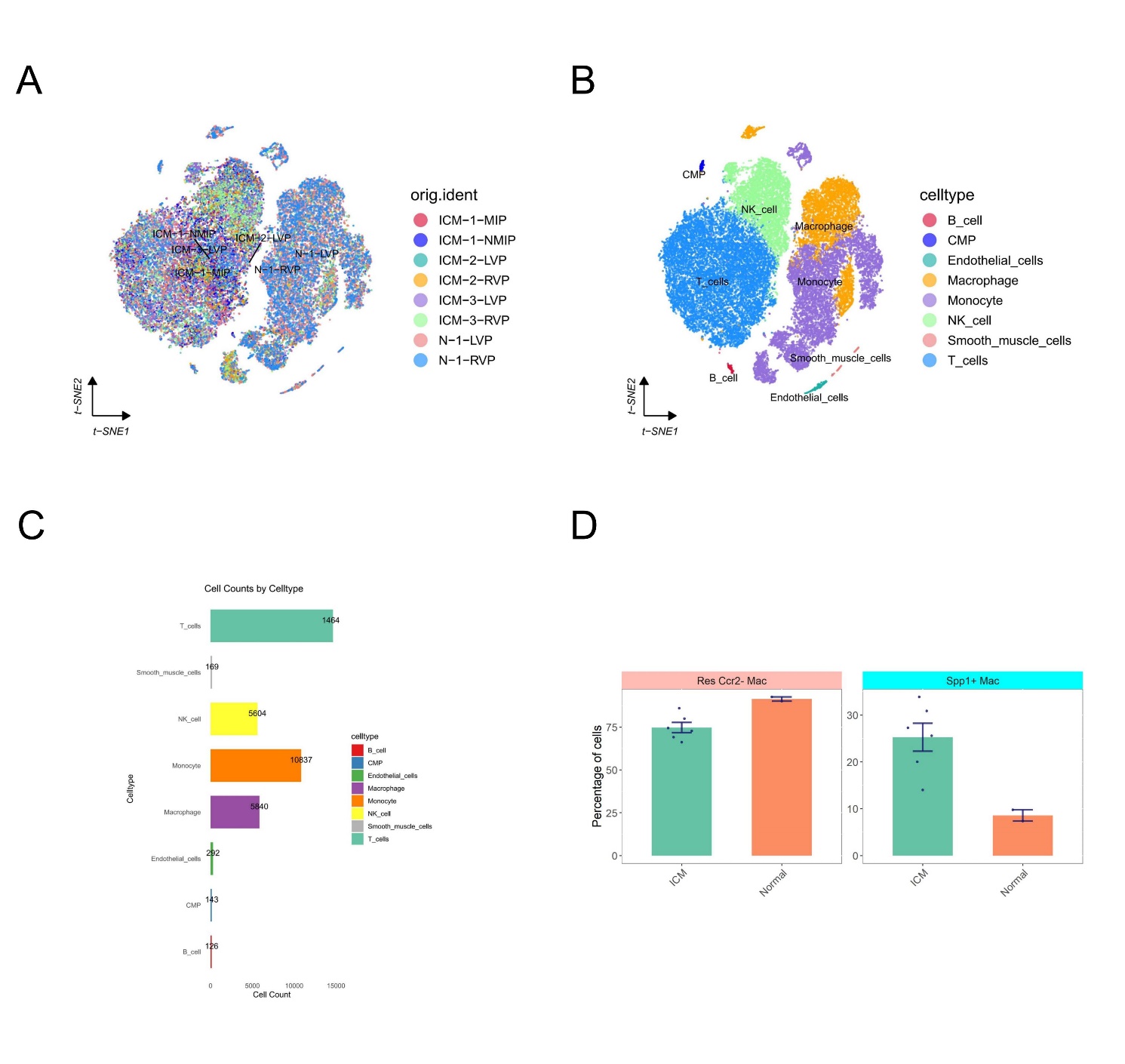


Supplementary Figure 4. Single-cell RNA sequencing analysis of cardiac cell populations under various conditions. (A) t-SNE plot demonstrating the distribution of cells from different experimental groups, with each dot representing a single cell and colors indicating the group identity, including various stages of induced cardiomyopathy (ICM) and normal (N) conditions. (B) t-SNE plot with cells color-coded by identified cell type, highlighting the diversity of cell populations within the heart, including macrophages, T cells, NK cells, and others. (C) Bar graph showing the cell counts for each identified cell type, with a clear predominance of certain cell populations. (D) Comparative bar graphs illustrating the percentage of resident Ccr2- macrophages and Spp1+ macrophages under control and induced (Injured) conditions, revealing shifts in population dynamics in response to cardiac stress.
